# Five Questions on Prion Diseases

**DOI:** 10.1371/journal.ppat.1002651

**Published:** 2012-05-03

**Authors:** Adriano Aguzzi, Caihong Zhu

**Affiliations:** Institute of Neuropathology, University Hospital of Zürich, Zürich, Switzerland; Washington University School of Medicine, United States of America

## Introduction

Prion diseases are characterized by deposition of PrP^Sc^, a misfolded and aggregated isoform of the host-encoded cellular prion protein (PrP^C^), within the central nervous system (CNS) and other organs. Here we review the current knowledge on five issues relevant to prion diseases: (1) how do prions enter the body, (2) how do prions reach the central nervous system, (3) how do prions damage the CNS, (4) do mammals have an antiprion defense system, and (5) how can the prion problem be resolved for good.

## How Do Prions Enter the Body?

Most cases of human prion disease occur for unknown reasons, and >20 mutations in the prion gene (*PRNP*) may lead to inherited prion disease. In other instances, prion diseases are contracted by exposure to prion infectivity. Save for direct brain exposure by neurosurgery, prions typically enter the body through extraneural pathways. This raises the intriguing question of how a mere protein aggregate can trespass mucosal barriers, circumvent innate and adaptive immunity, and travel across the blood–brain barrier to eventually provoke brain disease.

### Oral Uptake

There is no doubt that prion infections can be efficaciously transmitted under both natural and human-made conditions. While some aspects of the natural transmission of scrapie and chronic wasting disease (CWD) remain unexplained, oral transmission of prions has caused large epidemics and epizootics. Kuru, a human prion disease transmitted through ritual cannibalism, has afflicted the Fore people of Northern Papua New Guinea at extraordinarily high rates. Bovine spongiform encephalopathy (BSE), which has killed more than 280,000 cattle worldwide, is a prion disease caused by the feeding of recycled prion-infected foodstuff to cattle [1]. In turn, variant Creutzfeldt-Jacob Disease (vCJD), which has claimed over 200 victims, appears to be caused by consumption of BSE-contaminated beef products [2].

### Parenteral Uptake

Prions can also enter the body via parenteral uptake. Most worryingly, vCJD has been transmitted from subclinically vCJD-infected donors to recipients of transfused non-leukocyte-reduced red cells and of purified factor VIII preparations [3]. By far the largest incident involving iatrogenic prion transmission involved human pituitary hormones. Before the advent of recombinant DNA technology, growth hormone (used to treat dwarfism) and fertility hormones were recovered from human cadaveric pituitary glands. The prevalence of subclinically CJD-affected donors, probably coupled with brain-tissue contamination of pituitary extracts, led to over 160 prion deaths in mostly young recipients. Experimentally, the parenteral route of prion transmission is very effective and, e.g., intraperitoneal (i.p) inoculation of prions to laboratory animals is a widely used route for studies of peripheral prion replication and neuroinvasion.

### Intracerebral Administration

The most efficient way of prion transmission is intracerebral (i.c) administration. This is not unexpected, since the brain is the main target of prion toxicity and administration to the brain bypasses all natural barriers to prion neuroinvasion, such as the innate and adaptive immune system, as well as the blood–brain barrier.

Several iatrogenic CJD (iCJD) transmissions occurred via neurosurgery and dura mater grafts. These cases were particularly tragic for all those involved because they were inadvertently caused by medical personnel and represented an untoward effect of the intent to treat other ailments. Some of the first well-documented instances of intracerebral prion transmission to humans occurred in Zürich in the 1970s [4]. Electrodes were used for stereotactic electroencephalographic (EEG) recordings in a CJD patient, and reused after sterilization with ethanol and formaldehyde vapors—a procedure that reliably inactivates viruses and bacteria, but is ineffective against prions. This resulted in fatal prion transmission to two young patients. The infectivity on these electrodes was later confirmed by transmission of CJD to a chimpanzee.

### Aerosols

Prion transmission is usually not considered to be airborne like influenza or chicken pox. But we and others recently have found that prions can also be efficiently transmitted to mice through aerosols [5], [6]. Although aerosol-transmitted prions have never been found under natural conditions, this finding highlights the necessity of revising the current prion-related biosafety guidelines and health standards in diagnostic and scientific laboratories being potentially confronted with prion-infected materials.

## How Do Prions Reach the Central Nervous System?

### Role of B Cells

**Figure 1:**
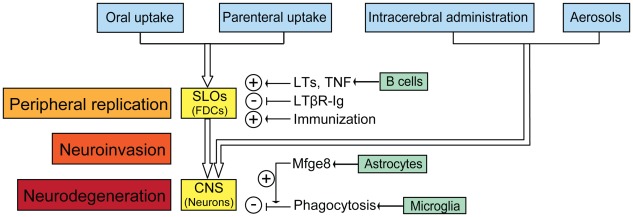
The cascade of prion entry, peripheral replication, neuroinvasion, and neurodegeneration. After peripheral exposure, prions colonize and replicate in secondary lymphoid organs (SLOs) like spleen, Payer's patches, lymph nodes, and tonsils. FDCs are the main sites accumulating prions in SLOs. B cell-derived LTs and TNF facilitate prion accumulation by supporting development and maintenance of FDCs. Dedifferentiation of FDCs by LTβR-Ig delays neuroinvasion, whereas repetitive immunization accelerates prion pathogenesis. Prions reach the central nervous system (CNS) through autonomic nerves, directly after intracerebral inoculation, or via aerosols through immune-independent pathways. In the brain, prions replicate but are also cleared by microglia after opsonisation by astrocyte-borne Mfge8. Prion deposition comes about when PrP^Sc^ production exceeds PrP^Sc^ clearance.

Prior to invading the CNS, prions frequently colonize in lymphoid organs (Fig. 1), where they colocalize with follicular dendritic cells (FDCs). The maintenance of FDCs depend on B cell-derived lymphotoxins (LTs) and tumor necrosis factor (TNF); accordingly, B cell–deficient mice (μMT, Rag1^−/−^, Rag2^−/−^) that lack mature FDCs are resistant to extraneural prion challenge [7]. However, the expression of PrP^C^ in B cells is dispensable, and transgenic expression of PrP^C^ restricted to B cells cannot restore prion replication or neuroinvasion in *Prnp* knockout mice. Therefore, B cells may function in an indirect way in prion diseases. It is reasonable to envision that B cells secrete factors (LTs, TNFs, etc.) that facilitate the maturation of cells like FDCs to replicate/accumulate prions.

### Role of Follicular Dendritic Cells

The immune system plays an important role in prion pathogenesis, but the exact nature of the cells replicating prions extraneurally is still unclear. FDCs are usually considered to be the main sites accumulating prions. Prion replication in lymphoid organs depends on PrP^C^-expressing FDCs, at least for the ME7 prion strain [8]. However, TNF receptor 1 knockout (TNFR1^−/−^) mice that lack mature FDCs are fully susceptible to peripheral prion infection and develop high prion titers in lymph nodes [9]. Furthermore, inflammatory granulomas that lack FDCs can also replicate prions in a lymphotoxin-dependent manner [10]. These results indicate cells other than FDCs are able to replicate prions extraneurally.

### Role of Autonomic Nerves

After replication and accumulation in lymphoid organs, prions invade the nervous system through sympathetic and parasympathetic nerves [11], [12]. The spread pathways of prions were determined by identifying the location and temporal sequence of pathological accumulation of PrP after oral challenge [11]. Upon i.p inoculation, permanent or transient sympathectomy chemically or immunologically delays or even prevents scrapie, whereas sympathetic hyperinnervation accelerates prion pathogenesis [12]. Hence innervation of lymphoid organs is rate-limiting for prion neuroinvasion. Furthermore, neuroinvasion velocity depends on the distance between FDCs and splenic nerves, suggesting that the neuroimmune transition of prions occurs between FDCs and sympathetic nerves.

## How Do Prions Damage the CNS?

The details of how prions induce toxicity are still unclear. Canonical caspase-mediated apoptosis is unlikely to be important, yet other pathways of cell death have remained largely unexplored. However, all attempts at a rational therapy necessitate a thorough understanding of how prions bring about the horrendous damage seen in spongiform encephalopathies.

### PrP^C^: An Amyloid Receptor?

PrP^C^ expression is indispensable for prion-induced neurotoxicity [13], implying PrP^C^ could be a receptor for prions to trigger detrimental signaling. The scenario could be broader. Strittmatter reported that PrP^C^ transduces the synaptic toxicity of amyloid-β (Aβ) oligomers in vitro [14]and in Aβ transgenic mice (APPswe/PSen1ΔE9) [15]. Moreover, different anti-PrP antibodies or their antigen-binding fragments that disrupt the PrP–Aβ interaction were able to block the Aβ-mediated disruption of synaptic plasticity. These findings were deemed exciting because they suggest the involvement of PrP^C^ in Alzheimer's disease (AD) pathogenesis. However, others found that the absence of PrP^C^ did not prevent deficits in hippocampal-dependent behavioral tests upon intracerebral Aβ injection [16]. Even more troubling was the report by Malinow [17] that Strittmatter's results could not be reproduced in a virtually identical paradigm. It has been suggested by Gerald Zamponi that variations in copper availability may contribute to these discrepancies.

We also crossed mice lacking or overexpressing PrP^C^ to the APPPS1 (APP^KM670/671NL^/PS1^L166P^) transgenic mice, yet did not see any effect of PrP^C^ on the impairment of hippocampal synaptic plasticity [18]. To make things even more confusing, another AD mouse model disproved any impact of PrP^C^ on Aβ-mediated neurotoxicity, whereas other studies appear to indirectly support the Strittmatter findings. Consequently, the question of whether PrP^C^ is a transducer of amyloid toxicity remains essentially unanswered. The discrepancies listed above do not necessarily result from any blatant flaws in the studies performed thus far, but rather indicate that some parameters affecting amyloid toxicity may still be unknown and, consequently, beyond the reach of rigorous testing.

### Shmerling's Disease and Baumann's Disease

Interstitially deleted PrP^C^ variants (Δ32–134; Δ94–134) elicit spontaneous neurodegeneration (Shmerling's and Baumann's disease), which is rescued by co-expression of full-length PrP^C^ [19], [20]. This suggests that truncated PrP^C^ competes with PrP^C^-like molecules through a shared receptor [20], [21]. Transgenic mice expressing deletion extended to the very end of N-terminus of PrP^C^ (Δ23–134) are healthy, suggesting that residues 23–31 are involved in Shmerling's and Baumann's disease [22].

Perhaps the key to understanding toxicity is the relationship between the N terminal tail and the plasma membrane. The amino terminus of full-length PrP^C^ is kept away from the membrane by the bulky globular domain. Perhaps the toxic mutants engage in deleterious interactions between the amino terminus and the membrane, possibly resulting in the formation of toxic pores [23].

### Lessons from Human Mutations

Transgenic mice expressing PrP^C^ with 14 octapeptide repeats insertion (PG14) in both *Prnp* ^+/+^ and *Prnp* ^−/−^ background displayed neurodegeneration similar to the disease of humans affected by a similar mutation [24]. In contrast to the syndromes described above, this pathology was not rescued by coexpression of PrP^C^, suggesting that the supernumerary octapeptides repeat expansion of PG14 PrP might induce neurodegeneration through another pathway distinct from Shmerling's and Baumann's disease. Accordingly, Shmerling's and Baumann's disease may arise through an allosteric mechanism, with the globular domain exerting its influence on the geometry of the amino terminal tail, whereas PG14 disease may be directly elicited by octapeptide repeats.

## Do Mammals Have an Antiprion Defense System?

### Evidence for Rapid Prion Clearance In Vivo

Progressive accumulation of PrP^Sc^ can only occur if conversion of PrP^C^ into PrP^Sc^ is faster than PrP^Sc^ clearance. Therefore, studying the clearance of prions is arguably as important as studying their generation. *Prnp* ^−/−^ mice develop more or less normally yet cannot replicate prions, making them a perfect model to study the half-life of the prion. Upon inoculation, residual infectivity all but disappears within 4 days, indicating that prions—commonly regarded as the sturdiest pathogens on earth—can be cleared in vivo with astonishing efficiency and speed. The identification of molecules and cells involved in prion clearance will be of great importance for therapeutics of prion diseases.

### Prion Clearance: Extracellular Proteolysis or Phagocytosis?

Neprilysin is a metalloprotease known to degrade extracellular amyloid such as Aβ. However, mice lacking or overexpressing neprilysin experience no changes in prion pathogenesis. Therefore, prion clearance may be effected by extracellular proteases other than neprilysin, or by different mechanisms altogether [25]. In organotypic cerebellar slices, the pharmacogenetic ablation of microglia led to a 15-fold increase in prion titers [26], suggesting that microglia are the primary effector of prion clearance.

### The Role of Mfge8

Microglia disposes of CNS debris (possibly including supernumerary synapses) by phagocytosis. But how can microglia identify prions as edible material? Milk fat globule epidermal growth factor 8 (Mfge8), a bridging molecule mediating phagocytosis of apoptotic cells, may represent a crucial link. Mfge8^−/−^ mice showed accelerated prion pathogenesis, accompanied with reduced clearance of cerebral apoptotic bodies and increased PrP^Sc^ accumulation and prion titers [27], suggesting Mfge8-mediated prion clearance in prion-infected mouse brain. More interestingly, these were observed in C57BL/6×129Sv but not in C57BL/6 genetic background. Therefore, besides Mfge8, other molecules involved in phagocytosis of apoptotic cells could have the potential to clear prions in vivo, it is worth putting more efforts on them.

## How Can the Prion Problem Be Resolved for Good?

### PrP^C^-Deficient Farm Animals: Technology and Hurdles

The absence of PrP^C^ is the only absolute guarantee that an organism will resist prion infections. Therefore it is of great practical interests to generate PrP^C^-deficient farm animals. Although it is unknown how tasty PrP^C^-deficient steaks might be, such farm animals would provide perfectly prion-free resources for all kinds of biologicals—including cytokines, growth factors, and therapeutic antibodies. Because embryonic stem cells are not available for gene targeting in other species, knockout farm animals were obtained by gene targeting of somatic cells followed by nuclear transfer. The first attempt to knock out *PRNP* in sheep was reported in 2001, but the cloned *PRNP* ^+/−^ sheep perished soon after birth—probably because of defective cloning procedures. In 2007, viable *PRNP* knockout cattle were obtained by sequential gene targeting in somatic cells and nuclear transfer. Targeted disruption of *PRNP* in goats, which frequently suffer from the prototypic prion disease, scrapie, was accomplished through a similar strategy [28], [29].

### Usefulness of PrP^C^-Deficient in Production of Biologicals

Biologicals are accounting for an ever increasing fraction of all therapeutics—yet all eukaryotically produced biologicals bear a certain risk of prion contamination, even when generated in cell lines. The transmission of vCJD through blood and even purified blood products has dramatically highlighted the seriousness of this threat. Therefore, PrP^C^-deficient farm animals (cattle and goats) are well positioned for the production of prion-free therapeutics and will therefore make an important contribution towards eliminating the risk of prions contamination in biologicals.

## Supporting Information

Table S1 Further reading
(DOC)
