# Supplementary material for: Five Questions on Prion Diseases
Source: PLoS Pathog. 2012 May 3;8(5):e1002651. doi: 10.1371/journal.ppat.1002651 (PMC3342938; doi:10.1371/journal.ppat.1002651)
Supplement: Table S1 — Further reading (DOC) [file ppat.1002651.s001.doc]

**Further reading list**

1. Gajdusek DC, Zigas V (1957) Degenerative disease of the central nervous system in New Guinea; the endemic occurrence of kuru in the native population. N Engl J Med 257: 974-978.

**This report describes the endemic occurrence of Kuru in Northern Papua New Guinea.**

2. Brown P, Preece MA, Will RG (1992) "Friendly fire" in medicine: hormones, homografts, and Creutzfeldt-Jakob disease. Lancet 340: 24-27.

**This paper reviews iatrogenic Creutzfeldt-Jakob disease caused by various medical manipulations.**

3. Stitz L, Aguzzi A (2011) Aerosols: An underestimated vehicle for transmission of prion diseases? Prion 5.

**The review discusses the context of aerosols transmission of prion disease and its impact on public health.**

4. Fraser H, Dickinson AG (1970) Pathogenesis of scrapie in the mouse: the role of the spleen. Nature 226: 462-463.

**The study showed for the first time that prions colonize secondary lymphoid organs in experimentally infected mice.**

5. Kitamoto T, Muramoto T, Mohri S, Doh-Ura K, Tateishi J (1991) Abnormal isoform of prion protein accumulates in follicular dendritic cells in mice with Creutzfeldt-Jakob disease. J Virol 65: 6292-6295.

**The paper showed the FDCs in lymphoid tissue are the sites of prion accumulation in mice infected with CJD prion.**

6. Klein MA, Frigg R, Raeber AJ, Flechsig E, Hegyi I, et al. (1998) PrP expression in B lymphocytes is not required for prion neuroinvasion. Nat Med 4: 1429-1433.

**This study revealed that the expression of PrP in B cells is not necessary for prion replication and neuroinvasion.**

7. Montrasio F, Cozzio A, Flechsig E, Rossi D, Klein MA, et al. (2001) B lymphocyte-restricted expression of prion protein does not enable prion replication in prion protein knockout mice. Proc Natl Acad Sci U S A 98: 4034-4037.

**This paper shows that B-cell expression of PrP alone cannot restore prion replication.**

8. McCulloch L, Brown KL, Bradford BM, Hopkins J, Bailey M, et al. (2011) Follicular Dendritic Cell-Specific Prion Protein (PrP) Expression Alone Is Sufficient to Sustain Prion Infection in the Spleen. PLoS Pathog 7: e1002402.

**This study found that FDC-specific expression of PrP can sustain prion (ME7 strain) replication in spleen, while depletion of PrP on FDC blocked the replication.**

9. Prinz M, Heikenwalder M, Junt T, Schwarz P, Glatzel M, et al. (2003) Positioning of follicular dendritic cells within the spleen controls prion neuroinvasion. Nature 425: 957-962.

**This paper showed that the neuroinvasion velocity of prion depends on the distance between FDCs and splenic nerves, and the neuroimmune transition of prions occurs between FDCs and sympathetic nerves.**

10. Steele AD, King OD, Jackson WS, Hetz CA, Borkowski AW, et al. (2007) Diminishing apoptosis by deletion of Bax or overexpression of Bcl-2 does not protect against infectious prion toxicity in vivo. J Neurosci 27: 13022-13027.

11. Steele AD, Hetz C, Yi CH, Jackson WS, Borkowski AW, et al. (2007) Prion pathogenesis is independent of caspase-12. Prion 1: 243-247.

**These two papers suggest that canonical caspase-mediated apoptosis is not the main pathway by which prions induce neurotoxicity.**

12. Chung E, Ji Y, Sun Y, Kascsak RJ, Kascsak RB, et al. (2010) Anti-PrPC monoclonal antibody infusion as a novel treatment for cognitive deficits in an Alzheimer's disease model mouse. BMC Neurosci 11: 130.

13. Barry AE, Klyubin I, Mc Donald JM, Mably AJ, Farrell MA, et al. (2011) Alzheimer's disease brain-derived amyloid-beta-mediated inhibition of LTP in vivo is prevented by immunotargeting cellular prion protein. J Neurosci 31: 7259-7263.

14. Freir DB, Nicoll AJ, Klyubin I, Panico S, Mc Donald JM, et al. (2011) Interaction between prion protein and toxic amyloid beta assemblies can be therapeutically targeted at multiple sites. Nat Commun 2: 336.

**These three papers claim that several anti-PrP antibodies are able to disrupt the interaction of PrP-Aβ and block Aβ-mediated disruption of synaptic plasticity.**

15. Cisse M, Sanchez PE, Kim DH, Ho K, Yu GQ, et al. (2011) Ablation of cellular prion protein does not ameliorate abnormal neural network activity or cognitive dysfunction in the J20 line of human amyloid precursor protein transgenic mice. J Neurosci 31: 10427-10431.

**This paper finds that PrP is not involved in Aβ-mediated neurodegeneration in the J20 mouse model of Alzheimer’s disease.**

16. Kudo W, Lee HP, Zou WQ, Wang X, Perry G, et al. (2011) Cellular Prion Protein Is Essential for Oligomeric Amyloid-beta-Induced Neuronal Cell Death. Hum Mol Genet.

17. Bate C, Williams A (2011) Amyloid-beta-induced synapse damage is mediated via cross-linkage of cellular prion proteins. J Biol Chem 286: 37955-37963.

**These two papers support Strittmatter’s claim that PrP is the mediator of Aβ-induced neurotoxicity.**

18. Bueler H, Fischer M, Lang Y, Bluethmann H, Lipp HP, et al. (1992) Normal development and behaviour of mice lacking the neuronal cell-surface PrP protein. Nature 356: 577-582.

**This work established the first line of *Prnp* knockout mice, and found that these mice develop and behave normally.**

19. Bremer J, Baumann F, Tiberi C, Wessig C, Fischer H, et al. (2010) Axonal prion protein is required for peripheral myelin maintenance. Nat Neurosci 13: 310-318.

**This paper revealed the role of PrP in peripheral myelin maintenance.**

20. Bueler H, Aguzzi A, Sailer A, Greiner RA, Autenried P, et al. (1993) Mice devoid of PrP are resistant to scrapie. Cell 73: 1339-1347.

21. Sailer A, Bueler H, Fischer M, Aguzzi A, Weissmann C (1994) No propagation of prions in mice devoid of PrP. Cell 77: 967-968.

**These two papers showed *Prnp* deficient mice are resistant to prion infection, cannot propagate prions, and that prions are cleared efficiently by these mice.**

22. Denning C, Burl S, Ainslie A, Bracken J, Dinnyes A, et al. (2001) Deletion of the alpha(1,3)galactosyl transferase (GGTA1) gene and the prion protein (PrP) gene in sheep. Nat Biotechnol 19: 559-562.

**This paper reported for the first time the succesful gene targeting in farm animals.**

23. Richt JA, Kasinathan P, Hamir AN, Castilla J, Sathiyaseelan T, et al. (2007) Production of cattle lacking prion protein. Nat Biotechnol 25: 132-138.

**This paper reported the generation of *Prnp*-deficient cattle.**

24. Yu G, Chen J, Xu Y, Zhu C, Yu H, et al. (2009) Generation of goats lacking prion protein. Mol Reprod Dev 76: 3.

**This paper reported the generation of *Prnp*-deficient goats.**

25. Montrasio F, Frigg R, Glatzel M, Klein MA, Mackay F, et al. (2000) Impaired prion replication in spleens of mice lacking functional follicular dendritic cells. Science 288: 1257-1259.

26. Mabbott NA, Mackay F, Minns F, Bruce ME (2000) Temporary inactivation of follicular dendritic cells delays neuroinvasion of scrapie. Nat Med 6: 719-720.

**This two papers showed that dedifferentiation of FDC impaired prion replication in mouse spleen, suggesting a therapeutic potential in prion diseases**.

27. Bremer J, Heikenwalder M, Haybaeck J, Tiberi C, Krautler NJ, et al. (2009) Repetitive immunization enhances the susceptibility of mice to peripherally administered prions. PLoS One 4: e7160.

**This paper showed that repetitive immunization accelerates prion pathogenesis.**
